# What Are the Restraint Practices, Preferences, and Experiences When Australian Parents Travel with Their Children in a Rideshare Vehicle?

**DOI:** 10.3390/ijerph18178928

**Published:** 2021-08-25

**Authors:** Sjaan Koppel, Sujanie Peiris, Mohammed Aburumman, Chernyse W. R. Wong, Justin M. Owens, Katie N. Womack

**Affiliations:** 1 Monash University Accident Research Centre, Monash University, Melbourne 3800, Australia; sujanie.peiris@monash.edu (S.P.); mohammed.aburumman@monash.edu (M.A.); chernyse.wong@monash.edu (C.W.R.W.); 2 Division of Vehicle, Driver and Safety Systems, Virginia Tech Transportation Institute, Blacksburg, VA 24060, USA; jowens@vtti.vt.edu; 3 Center for Transportation Safety, Texas A&M Transportation Institute, College Station, TX 77843, USA; K-Womack@tti.tamu.edu

**Keywords:** rideshare services, child occupant, child restraints, restraint, parents, road safety

## Abstract

This study aimed to explore the preferences, experiences and restraint practices of Australian parents travelling with their children in rideshare vehicles. Six hundred and thirty-one participants completed an online survey (M = 39.2 years, SD = 10.5, Range = 18.0–70.0 years; Female: 63.4%). Most participants (59.1%) reported that they had not travelled in a rideshare vehicle with their youngest child (M = 7.2 years, SD = 5.2, Range = 0.0–17.0 years; Male: 54.2%). Participants who reported that they have travelled with their youngest child in a rideshare vehicle tended to: be younger, identify as male, have completed an Undergraduate or Postgraduate degree, reside in the Australian Capital Territory, earning a higher yearly household income, and were involved in an at-fault crash in the past two years. In addition, these participants were: less likely to have a ‘younger’ youngest child, less likely to ‘always’ wear a seatbelt while travelling in their private motor vehicle, and also less likely to ‘always’ restrain their child in an appropriate restraint while travelling in their private motor vehicle. Prohibitive reasons for not travelling in a rideshare vehicle included: cost (29.3%), concerns over driver safety (27.5%), concerns over travelling with children in a rideshare service (24.8%), or inconvenience (24.3%). Participants who reported that they had travelled in a rideshare vehicle with their youngest child reported lower rates of appropriate restraint use within the rideshare vehicle (57.3%) than when travelling in their private motor vehicle (85.6%). Reasons associated with inappropriate restraint use within the rideshare vehicle included: unavailability of a child restraint (39.6%), travelling a short distance (33.0%), were not required to use one in this situation (33.0%), or the parent did not have a restraint with them (26.4%). Given the increasing popularity of rideshare services in Australia, and globally, the urgent adaption of rideshare-specific policy, legislation, education, and design in relation to child restraint requirements is needed to ensure the safety of child occupants.

## 1. Introduction

In Australia, rideshare services are defined as a service where an individual can organise or hire a personal driver to take them exactly where they need to go without sharing the vehicle with any other individuals, nor having to make any other stops along a route. These services have experienced a dramatic rise in popularity in Australia, and other industrialised countries over the past few years [1]. Given the global emphasis on safe, affordable, accessible, and sustainable transportation modes [2], the use of rideshare services is predicted to increase significantly [3]. For example, the proportion of Australians using the ‘Uber’ rideshare service across an average three-month period increased from 6.6 percent of the population in 2016 to 22.9 percent in 2019 [4]. This increase in usage of rideshare vehicles parallels the decline of driver licence uptake in Australia. In addition, the average age of individuals applying for a driver’s licence has increased, suggesting that, for younger generations, the private motor vehicle does not hold the same importance as it once did [1]. Recent findings [5] have also indicated that parents who lived in urban settings were more likely to use rideshare vehicles than parents in non-urban environments. These trends are likely to have serious implications for child-occupant safety as families use rideshare vehicles as a family transportation option [6].

Despite recent advances in motor vehicle and child restraint design, motor vehicle crashes remain the leading cause of child death in Australia [7]. Existing evidence suggests that child restraints offer a high level of crash protection during an impact, potentially reducing injury by 70 percent compared with unrestrained children [8,9,10]. However, the effectiveness of the child restraint is critically dependent on the correct installation of the restraint within the vehicle, correct harnessing of the child within the restraint, and the use of an appropriate restraint for the child’s size. Incorrect and/or inappropriate fitment and use of child restraints may reduce or nullify its safety benefits [11,12].

Although child restraint use is high (i.e., over 90%) in private motor vehicles in Australia [13,14,15], research has shown that child restraint use is substantially lower in shared transportation modes such as taxis, rideshare vehicles and carpooling [6,16,17,18,19]. For example, in the United States, Owens and colleagues [6] reported that 59 percent of parents restrained their children aged five years and younger ‘differently’ when travelling in a rideshare vehicle than they did when travelling in their private motor vehicle, including holding the child on their lap (37.0%) or letting their child travel without an appropriate child restraint (25.0%). The most frequently cited reasons for not using a child restraint while travelling in a rideshare vehicle included: the driver/vehicle did not have a child restraint, the participant did not have a child restraint, or the trip was a short distance. To date, the restraint practices, preferences, and experiences when Australian parents travel with their children in rideshare vehicles have not been explored.

Complicating matters, there are some differences across the Australian states and territories regarding their exemptions for child restraint requirements. In Australia, child restraint requirements across the states and territories are mainly equivalent to the requirements stated in Australian Road Rules [20] (see Supplementary Table S1).

Specifically:

- Child occupants aged less than six months are required to be restrained in a rearward-facing child restraint;
- Child occupants aged between six months and four years are required to be restrained in either a rearward-facing child restraint or a forward-facing child restraint with an inbuilt harness.
- Child occupants aged between four and seven years are required to be restrained in either a forward-facing child restraint with an inbuilt harness or a booster seat that is restrained by either a lap and sash type seatbelt or by a child safety harness. However, some states provide concessions to this requirement. More specifically:
  ○ In the state of Victoria, the requirements also allow for child occupants in this age group to occupy a seating position that is fitted with a seatbelt and restrained in either a lap and sash type seatbelt or a lap type seatbelt equipped with a child safety harness.
  ○ In the state of Western Australia, the requirements allow for the booster seat to be restrained by either a lap and sash type seatbelt, or a lap-only type seatbelt and a child safety harness.
- Child occupants aged seven years and older are required to be restrained by either a lap and sash type seatbelt or a lap-only type seatbelt.

However, the Northern Territory, Queensland and Western Australia allow rideshare vehicles to be exempt from the child restraint requirements. Other states and territories, for the most part, also allow taxis to be exempt from these requirements, except for New South Wales, where taxis are not exempt from the requirements that apply to child occupants aged less than 12 months. Other transport modes exempt from these child restraint requirements in some states and territories include public minibuses, hire cars and tow trucks.

Given the anticipated increase in the use of rideshare vehicles in Australia, and that child restraint use is likely to be lower in this mode of transport, this study aimed to explore the restraint practices, preferences, and experiences when Australian parents travel with their children in rideshare vehicles.

## 2. Materials and Methods

### 2.1. Participants

Participants were eligible to participate if they: (a) resided in Australia, (b) were aged 18 years and older; (c) were an ‘active’ driver (i.e., at least once per week in the pre-COVID period), and (d) had at least one child (aged 17 years or younger) who currently lives with them.

### 2.2. Materials

Participants completed an online survey (approximately 25 min, see Supplementary File S1) which is described below.

#### 2.2.1. Socio-Demographic Characteristics

Participants provided information about their: age, gender, marital status, the highest level of completed education, current annual household income and residential state or territory.

#### 2.2.2. Driving and Licensing Characteristics

Participants provided information on their licensing history, annual mileage, frequency of driving (where: 1 = Daily; 5 = Less than once per week), previous crash involvement and/or driving infringements, and frequency of wearing their seatbelt while travelling in a motor vehicle (where: 1 = Always, 6 = Never).

#### 2.2.3. Child Characteristics and Their Travel Patterns

Participants were asked to provide information regarding the number (and the age) of any children living with them. Participants with more than one child were asked to answer the remaining sections of the survey in reference to their youngest child and provide information on the child’s: gender, the frequency with which the child travelled in a motor vehicle when the participant was the driver (where: 1 = Daily; 8 = Never), the type of restraint that the child used most often when the participant was the driver (i.e., rearward-facing child restraint, forward-facing child restraint, booster seat, seatbelt, no restraint), the frequency that the child used their restraint when the participant was the driver (where: 1 = Always; 6 = Never), and the location where the child sat within the motor vehicle when the participant was the driver (e.g., front passenger seat, rear seat, etc.). Participants were asked to provide information on the frequency with which they travelled with this child using other modes of transportation, including: as a passenger in someone else’s vehicle, as a pedestrian, as a cyclist, as a passenger on public transport (i.e., train, tram, or bus), as a passenger in a taxi, or as a passenger in a rideshare vehicle (where: 1 = Daily; 8 = Never).

#### 2.2.4. Restraint Practices, Preferences, and Experiences When Travelling with Children in a Rideshare Vehicle

Participants who indicated that they had travelled in a rideshare vehicle with their youngest child were asked to indicate the types of trips undertaken when travelling in a rideshare vehicle, the restraint used by their child when travelling in a rideshare vehicle, and details of the restraint used including: the confidence of child restraint installation correctness, reasons for non-use of child restraints or seatbelts in a rideshare vehicle (if applicable), and their familiarity with state laws pertaining to child restraints and seatbelt use.

#### 2.2.5. Non-Use of a Rideshare Vehicle When Travelling with Children

Participants who indicated that they have not travelled in a rideshare vehicle with their youngest child were asked to provide reasons for not using a rideshare vehicle.

### 2.3. Procedure

The study was approved by the Monash University Human Research Ethics Committee (MUHREC). Participants were recruited through various online and social media advertising, such as the MUARC Facebook page and Twitter feed and the Monash University Insider newsletter. The advertising directed participants to an online survey link. To improve recruitment, participants who completed the online survey were able to opt into a draw to win one of five $100 gift vouchers. The online survey was administered from August–November 2020.

### 2.4. Data Analysis

Descriptive statistics were conducted to describe the sample. A series of chi-square analyses were conducted to explore the differences between participants who reported that they had and had not travelled with their child(ren) in a rideshare vehicle. All statistical analyses were conducted using IBM SPSS v. 28.

## 3. Results

The findings for this study are presented in three main sections: (1) participants’ socio-demographic characteristics, as well as their driving and licensing history; (2) characteristics of the participants’ youngest child and their restraint and travel patterns; and (3) restraint practices, preferences, and experiences when Australian parents travel with their children in a rideshare vehicle.

### 3.1. Participants’ Socio-Demographic Characteristics

Six hundred and thirty-one participants completed the online survey. As shown in Table 1, the majority of participants: were aged between 25 and 34 years (32.2%; M = 39.2 years, SD = 10.5, Range = 18.0–70.0 years); were female (63.4%); were in a married/defacto relationship (85.9%); had completed an undergraduate degree (31.1%); had a yearly household income ($AUD) of between $75,001–100,000 before tax (17.7%); and lived in the Australian states of New South Wales or Victoria (30.6%, 29.5%, respectively).

### 3.2. Participants’ Driving and Licensing Characteristics

All participants were current drivers and held a valid driver’s licence, and 86.1% (*n* = 543) of participants reported that they did not have any licence conditions or restrictions. Many participants reported that: they drove daily (56.3%), had driven between 10,001 and 15,000 kilometres in their vehicle over the past year (23.8%), and ‘always’ wore their seatbelt while driving or travelling in a vehicle (92.6%, see Table 2). Over the past two years, many participants reported that they had not been involved in a motor vehicle crash (90.6%) or an at-fault crash (95.1%), had not been cited for failing to stop (95.6%), speeding (90.0%), or other driving infringements such as using a mobile phone illegally while driving (97.6%).

### 3.3. Participants’ Youngest Child Characteristics and Their Restraint and Travel Patterns

The majority of participants reported that they had one or two children currently living with them (1: 46.1%; 2: 38.8%; 3: 13.0%; 4: 1.6%; 5: 0.3%; 6: 0.2%). Many participants reported that their youngest child: was aged between one and three years (29.0%; M = 7.2 years, SD = 5.2, Range = 0.0–17.0 years); was male (54.2%); travelled in the vehicle with them between four to six times per week (38.8%), was restrained by a seatbelt (51.1%), was ‘always’ restrained (85.6%), and was seated in the rear seat (2nd or 3rd row) of the vehicle (74.3%) (see Table 3).

**Figure 1:**
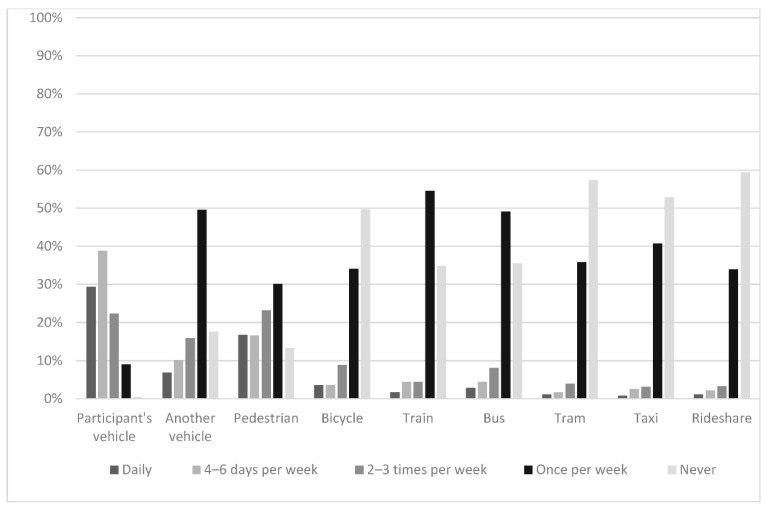
Participants’ use of different transportation modes with their youngest child.

Participants were asked to rate the frequency with which they have used different transportation modes with their youngest child (see Figure 1). Two-thirds of participants (68.1%) reported that they drove with their youngest child in their personal motor vehicle at least four days per week, while more than half of the participants reported that they ‘never’ used trams (57.3%), taxis (52.8%) or rideshare services (59.4%) with their youngest child.

### 3.4. Reasons That Australian Parents Have Not Travelled with Their Children in a Rideshare Vehicle

Participants who reported that they had ‘never’ travelled in a rideshare vehicle with their youngest child (*n* = 375, 59.4%) stated they had not done so for various non-exclusive reasons (participants were allowed to select more than one response, see Table 4). These reasons included that the rideshare vehicle was too expensive (29.3%), there were concerns over driver safety (27.5%) or travelling with children in a rideshare vehicle (24.8%), or because the service was not convenient (24.3%). The main ‘other’ reason for not using rideshare vehicles was that there was ‘no need/requirement’ because participants had their vehicle.

#### Child-Specific Reasons for Not Travelling in a Rideshare Vehicle

Participants who reported that they had ‘never’ travelled in a rideshare vehicle with their youngest child because of their concerns over travelling with children (*n* = 93), did so for various non-exclusive child-specific reasons (i.e., participants were allowed to select more than one response) (see Table 5). Participants were most likely to report that rideshare vehicles were not a ‘practical’ or ‘convenient’ option when travelling with children (57.0%, 49.5%, respectively). Participants also noted that they had not travelled in a rideshare vehicle with their youngest child because they required a child restraint or booster seat and; a) the participant did not have one with them, or b) it was not provided by the rideshare vehicle driver (45.2% for both). The main ‘other’ reason for not using rideshare vehicles was that they did not ‘trust’ the driver.

### 3.5. Restraint Practices, Preferences, and Experiences When Australian Parents Travel with Their Children in a Rideshare Vehicle

As noted above in Figure 1, 40.6 percent of participants reported travelling in a rideshare vehicle with their youngest child (*n* = 256) and reported that they did so across different situations and with different frequencies (see Table 6). For example, participants were most likely to have used a rideshare vehicle with their youngest child for travel during a holiday or out-of-town trip (90.2%) and least likely to use a rideshare vehicle for routine local travel where they live (76.6%).

The majority of participants who had used a rideshare vehicle with their youngest child also reported that their child was ‘always’ restrained (57.3%), was seated in the rear seat (2nd or 3rd row) of the rideshare vehicle (80.3%) and was most likely to be restrained by a seatbelt during the journey within the rideshare vehicle (43.1%) (see Table 7). It should be noted that a small proportion of participants had not used a rideshare service to travel with their youngest child in these four situations listed in Table 6 over the past two years (3.1%, *n* = 8).

Participants who reported that they did not ‘always’ use an appropriate restraint (i.e., according to restraint requirements stated in Australian Road Rules [20]) in a rideshare vehicle for their youngest child did so for various non-exclusive reasons (i.e., participants were allowed to select more than one response, see Table 8). The most frequent responses were that the driver did not have a child restraint available (39.6%), that they were travelling a short distance (33.0%), that they were not required to use one in this situation (33.0%), or the parent did not have a restraint with them (26.4%). It was interesting to note that 9.4 percent of participants who reported that they did not ‘always’ use an appropriate restraint reported that they held their child in their lap.

Participants who reported that they had restrained their youngest child in a child restraint or booster seat (rearward-facing CRS: *n* = 44; forward-facing CRS: *n* = 60; booster seat: *n* = 26) while travelling in a rideshare vehicle in the past two years reported that they, as parents, were most likely to have: provided the child restraint or booster seat (74.6%), installed the child restraint or booster seat on their own (53.1%) and to have adjusted the harness or seatbelt in the child restraint or booster seat on their own (66.9%) (see Table 9). In addition, participants were most likely to report that they were ‘confident’ or ‘very confident’ that the child restraint or booster seat was installed correctly (45.4%, 40.8%, respectively). Finally, of the participants who had travelled with their child(ren) in a rideshare vehicle, most reported that they were ‘confident’ or ‘very confident’ that they had followed state or territory laws about child restraint use when travelling in a rideshare vehicle (43.1%, 39.2%, respectively).

### 3.6. Characteristics of the Australian Parents Who Have Travelled with Their Youngest Children in a Rideshare Vehicle

The characteristics of Australian parents who have travelled with their youngest child(ren) in a rideshare vehicle are presented in Table 10. Participants who reported that they have travelled in a rideshare vehicle with their youngest child tended to: be younger (aged 18–24 years), identify as male, have completed an Undergraduate or Postgraduate degree, reside in the Australian Capital Territory, earning a higher yearly household income, and to have been involved in an at-fault crash in the past two years. In addition, these participants were also: less likely to have a ‘younger’ youngest child, less likely to ‘always’ wear a seatbelt while travelling in their private motor vehicle, and less likely to ‘always’ restrain their child in an appropriate restraint while travelling in their personal motor vehicle.

## 4. Discussion

This study provides new insights regarding the restraint practices, preferences, and experiences of Australian parents when they travel with their children in a rideshare vehicle. This is an important area of research given that: (1) the use of rideshare vehicles in Australia is significantly increasing, (2) motor vehicles crashes are the leading cause of death for Australian children, and (3) correct and appropriate child restraint use is substantially lower in this mode of transport, thereby increasing the risk of death or serious injury for child occupants in the event of a motor vehicle crash.

Most participants in this study reported that they had not travelled in a rideshare vehicle with their youngest child (59.1%). Consistent with the findings of Owens and colleagues [6], the main reasons for not travelling in a rideshare vehicle were related to concerns regarding cost, driver safety, travelling with children, and convenience with rideshare vehicles. Similarly, Ehsani [3] recently highlighted that parents may find the price a barrier when ridesharing with children, thus reducing their use of such services. The additional costs can arise from ordering rideshare vehicles with company-provided child restraints [3]; or needing to order a larger vehicle to accommodate additional passengers [6]. For example, Uber’s car seat program in the United States allows passengers to request a child restraint [6] for a USD $10 surcharge [21]. Considering the surcharge is one-third of the average trip fare of $30 [22], it could be a contributing factor to why parents have not used rideshare vehicles with children.

While there is significant ease or convenience in matching drivers to potential passengers in real-time via geolocation on smartphones, the ridesharing innovation has encountered some scrutiny [23]. With minimal government oversight present to protect rideshare passengers from driver error or abuse, vehicle failure or violence, rideshare vehicles are not exempt from crime, and the passenger’s safety is still a much-debated issue [24]. Despite scientifically rigorous research suggesting that ridesharing services provide improved passenger safety [25,26], particularly regarding the reduction of alcohol-related fatalities [26,27,28,29], there is an equal amount of evidence to suggest passenger safety is compromised in rideshare services due to driver abuse with sexual assaults, motor vehicle fatalities and fatal physical assaults [30,31,32].

Approximately 40 percent of participants in this study reported that they have travelled in a rideshare vehicle with their youngest child and were most likely to have done so during a holiday or out-of-town trip (90.2%). Participants who reported that they have travelled in a rideshare vehicle with their youngest child tended to: be younger (aged 18–24 years), identify as male, have completed an Undergraduate or Postgraduate degree, reside in the Australian Capital Territory, earning a higher yearly household income, and to have been involved in an at-fault crash in the past two years. In addition, these participants were also: less likely to have a ‘younger’ youngest child, less likely to ‘always’ wear a seatbelt while travelling in their private motor vehicle, and less likely to ‘always’ restrain their child in an appropriate restraint while travelling in their personal motor vehicle. These findings are consistent with prior studies, where rideshare use was higher among more educated and affluent segments of the population [3,33].

The Australian Automobile Association’s [34] quarterly *Transport Affordability Index* found that private car ownership for metropolitan households made up 13 percent of the household income or $344 per week on transport cost. The Australian Capital Territory (ACT) had the most affordable transport cost nationally, with household transport cost below the national average, at $320 per week. This may explain why participants residing in the ACT were most likely to have travelled in a rideshare vehicle with their youngest child. ACT has the second-highest median household income in Australia [35]; thus, when combined with low transport costs, individuals may have more disposable income and be more inclined to pay for utilising rideshare services. DA Economics [36] found that 64 percent of Uber trips in Sydney began or ended in a transport desert. Analogously, ACT may have similar transport deserts, which could account for the high number of rideshare users in this study.

One of the most important findings of this study is that participants reported lower rates of appropriate restraint use for their children when travelling in a rideshare vehicle compared to when travelling in their private motor vehicle. For example, while 85.6 percent of participants reported that they ‘always’ restrained their youngest child in an appropriate restraint while travelling in their private motor vehicle, only 57.3 percent of participants reported that they ‘always’ restrained their youngest child in an appropriate restraint when they travelled in a rideshare vehicle. These findings are consistent with several studies from the United States who have recently noted that parents restrain their children ‘differently’ while travelling in alternative transport modes such as rideshare vehicles [6,19], taxis [16,18], or carpooling [17]. These findings have significant implications, suggesting that child occupants may be at an increased risk of death or serious injury in the event of a motor vehicle crash while travelling in these modes of transport. Owens and colleagues [6] reported that 59 percent of parents restrained their children aged five years and younger ‘differently’ when travelling in a rideshare vehicle than they did when travelling in their private motor vehicle, including holding the child on their lap (37%) or letting their child travel without an appropriate child restraint (25%). The most frequently cited reasons for not using a child restraint while travelling in a rideshare vehicle included: the driver/vehicle did not have a child restraint, the participant did not have a child restraint, or the trip was a short distance. Similarly, we found that participants who reported that they did not ‘always’ use an appropriate restraint in a rideshare vehicle for their youngest child were most likely to do so did so because the driver did not have a child restraint available (39.6%), the parent did not have a restraint with them (33.0%), or they did not use a child restraint or booster seat because they believed one was not required (33.0%).

The findings from our study highlight the need for some state regulators to clarify regulations to address child restraint requirements in rideshare vehicles. For example, taxis are exempt from child restraint requirements in Victoria, whereas rideshare vehicles are not exempt from these requirements. These conflicting requirements could potentially be a source of confusion to Victorian parents and drivers of rideshare vehicles, as they might mistakenly believe they are not breaking the law if not using an appropriate child restraint in a rideshare vehicle. Moreover, the findings from our study highlight the need for more significant efforts towards improving awareness of both parents and drivers regarding local laws for child restraints in rideshare vehicles. Rideshare service providers could also play a proactive role in ensuring their drivers know and comply with local child restraint requirements by requiring CRS use according to private vehicle regulations. Appropriate CRS use in rideshare vehicles could also be encouraged by injury prevention practitioners and paediatricians [3].

Rideshare companies could also provide a child restraint program, like Uber [6], in partnership with state agencies and organisations like KidSafe, which checks and installs child restraint seats across Australia. Child restraint manufacturers could address the ease of use and portability of restraints [19], while car manufacturers could design an integrated child seat in their vehicles [37]. Moreover, our study highlighted a disproportionate use of rideshare vehicles among high-income earners, which raises the question of whether policy-makers and city planners need to consider equity of access to ridesharing for families with children and in general.

Several limitations should be noted. The findings from the current study are based on a convenience sample and may be the result of a volunteer bias (i.e., individuals who agreed to participate in the online survey may be more interested in rideshare vehicles or road safety in general). Similarly, the findings from the current study are based on self-reported behaviour. However, it should be noted that previous research has suggested that participants tend to minimise the extent or frequency of their behaviours if they are not considered to be socially acceptable (e.g., speeding, non-use of restraints) [38]. Future research could employ revealed preference methodologies to explore these issues. In addition, to investigate whether child restraint use is lower in rideshare vehicles than in private motor vehicles, we recruited active drivers. However, some participants may transport children who are not active drivers, which may have biased our results. Future research should explore these issues with a broader sample of Australian parents. In addition, to collect data systematically and more easily per household, participants were asked to report on the restraint practices, preferences, and experiences when travelling with their youngest child. It is possible that these results could be different if we asked participants to report on their eldest child. Our study compared most of our findings against Uber use in Australia, which limits global generalisability. While Uber is the largest rideshare service provider in Australia, data from other providers such as Didi, Ola, GoCatch have not been considered in this study; thus, extrapolations from our findings should consider this. Another reason participants cited for not using rideshare vehicles was that there was ‘no need’ because they had a personal vehicle. Clewlow and Mishra [39] found that people living in more urban metropolitan areas were three times more likely to use rideshare vehicles regularly than people living in suburban metropolitan areas. Future studies could explore the relationship between urban density, household structure, and the use of rideshare vehicles to investigate the reasoning behind parents’ transportation choices.

Additionally, ridesharing is often thought of as a solution to the ‘last mile’ problem [40], and environmental factors like an efficient public transport network and the availability of amenities and services possibly limit the usage of rideshare vehicles in isolation. Parents may be willing to utilise both rideshare and public transport to get “from their door to a transport hub” [40] (p. 9) and vice versa. However, a range of factors, such as the efficiency and punctuality of public transport and the interconnectedness of the public transport network, would need to be examined and considered to ascertain further the relationship between public infrastructure and the use of rideshare vehicles with children.

## 5. Conclusions

Given the dramatic rise in popularity of rideshare services in Australia and globally, child occupant safety policy and legislation, education, and child restraint design need to be adapted to ensure the safety of child occupants in every vehicle transportation mode.

## Figures and Tables

**Table 1:** Participants’ socio-demographic characteristics (*n* = 631).

	% (*n*)
Age (years)	
18–24	6.3% (40)
25–34	32.2% (203)
35–44	27.4% (173)
45–54	26.0% (164)
55+	8.1% (51)
Sex	
Male	36.6% (231)
Female	63.4% (400)
Marital Status	
Single	8.4% (53)
Married/Defacto	86.2% (544)
Separated/Divorced	4.3% (27)
Widowed	1.1% (7)
Highest level of completed education	
Primary/Intermediate (Year 10 equivalent)	5.9% (37)
High (Year 12 equivalent)	5.4% (34)
Technical/Trade (incl. apprenticeship)	17.3% (109)
Diploma	12.7% (80)
Undergraduate degree	31.1% (196)
Postgraduate degree	23.1% (146)
Yearly household income ($AUD) before taxes	
<$25,000	3.2% (20)
$25,001–$50,000	12.8% (81)
$50,001–$75,000	13.6% (86)
$75,001–$100,000	17.7% (112)
$100,001–$125,000	13.6% (86)
$125,001–$150,000	7.9% (50)
$155,001–$175,000	6.7% (42)
$175,001–$200,000	10.6% (67)
$200,001–$250,000	6.0% (38)
>$250,001	3.6% (23)
Prefer not to say	4.1% (26)
Residential state/territory	
Australian Capital Territory	2.7% (17)
New South Wales	30.6% (193)
Northern Territory	0.3% (2)
Queensland	18.4% (116)
South Australia	6.3% (40)
Tasmania	1.7% (11)
Victoria	29.5% (186)
Western Australia	10.5% (66)

**Table 2:** Participants’ driving characteristics (*n* = 631).

	% (*n*)
Frequency of driving	
Daily	56.3% (355)
4–6 times per week	31.5% (199)
2–3 times per week	9.5% (60)
<1 time per week	2.7% (17)
Estimated kms driven in their vehicle over the past year	
<5000 km	20.3% (128)
5001–10,000 km	22.8% (144)
10,001–15,000 km	23.8% (150)
15,001–20,000 km	15.1% (95)
20,001–25,000 km	9.4% (59)
>25,001 km	8.7% (55)
Frequency of wearing a seatbelt while travelling in a motor vehicle	
Always	92.6% (584)
Almost always/Usually Sometimes/Almost never	6.8% (43)
Never	0.6% (4)
Over the past two years, involved in a crash while driving (incl. minor crashes)?	
No	90.6% (572)
Yes	9.4% (59)
Over the past two years, involved in an at-fault crash while driving (incl. minor crashes)?	
No	95.1% (600)
Yes	4.9% (31)
Over the past two years, cited for failing to stop at a stop sign or traffic signal (including red light cameras)?	
No	95.6% (603)
Yes	4.4% (28)
Over the past two years, cited for speeding?	
No	90.0% (568)
Yes	10.0% (63)
Over the past two years, cited for other driving offences (e.g., using a mobile phone illegally)?	
No	97.6% (616)
Yes	2.4% (15)

**Table 3:** Participants’ youngest child characteristics, restraint, and travel patterns (*n* = 631).

	% (*n*)
Age group	
<1 year	5.2% (33)
1–3 years	29.0% (183)
4–7 years	23.0% (145)
8–12 years	22.5% (142)
13–17 years	20.3% (128)
Sex	
Male	54.2% (342)
Female	45.5% (287)
Other	0.3% (2)
Frequency of travelling in a motor vehicle	
Daily	29.3% (185)
4–6 times per week	38.8% (245)
2–3 times per week	22.3% (141)
Once per week	5.5% (35)
Less than once per week	2.7% (17)
Less than once per month	0.8% (5)
Never	0.5% (3)
Restraint type	
Rearward-facing CRS	11.3% (71)
Forward-facing CRS	22.3% (141)
Booster seat	21.7% (137)
Seatbelt	41.8% (264)
No restraint	2.9% (18)
Frequency of being restrained while travelling in a motor vehicle	
Always	85.6% (540)
Almost always/Usually/Sometimes	10.3% (65)
Never	4.1% (26)
Seating position within the vehicle	
Front passenger seat	25.0% (158)
Rear seat (back seat of vehicle, 2nd or 3rd row of a minivan)	74.3% (469)
Someone’s lap	0.6% (4)

**Table 4:** Participants’ reasons for not travelling in a rideshare vehicle with their youngest child (*n* = 375).

Reasons	% (*n*)
Too expensive	29.3% (110)
Concerns over driver safety	27.5% (103)
Concerns over travelling with children	24.8% (93)
Not convenient	24.3% (91)
Concerns over vehicle safety	15.7% (59)
Not available in my area	15.2% (57)
Not available when I needed it	7.5% (28)
No smartphone access	1.6% (6)
Other	23.5% (88)

**Table 5:** Participants’ child-specific reasons for not travelling in a rideshare vehicle with their youngest child (*n* = 93).

Reasons	% (*n*)
Not a practical option when travelling with children	57.0% (53)
Not a convenient option when travelling with children	49.5% (46)
Child required a child restraint or booster seat and I didn’t have one with me	45.2% (42)
Child required a child restraint or booster seat and it wasn’t provided by the driver	45.2% (42)
Not a safe option when travelling with children	41.9% (39)
Extra cost for a child with a child restraint or booster seat was too expensive	18.3% (17)
Too many passengers for rideshare service vehicle	5.4% (5)
Other	5.4% (5)

**Table 6:** Situations (and frequency) that participants used rideshare services to travel with their youngest child over the past two years (*n* = 256).

Situations	Regularly (>10 Trips) % (*n*)	Often (6–10 Trips) % (*n*)	Occasionally (1–5 Trips) % (*n*)	Never (0 Trips) % (*n*)
For local travel during a holiday/out-of-town trip (e.g., airport to hotel, hotel to restaurant)	21.5% (55)	18.0% (46)	50.8% (130)	9.8% (25)
For routine local travel where they live (e.g., daily activity, school drop off or pickup, shopping, social activities)	12.9% (33)	19.9% (51)	43.8% (112)	23.4% (60)
For non-routine local travel where they live (e.g., usual vehicle not available, emergencies)	8.6% (22)	20.3% (52)	52.3% (134)	18.8% (48)
To travel to a holiday/out-of-town trip destination	11.3% (29)	16.8% (43)	50.4% (129)	21.5% (55)

**Table 7:** Participants’ youngest child’s restraint in a rideshare vehicle over the past two years (*n* = 248).

	% (*n*)
Restraint type	
Rearward-facing CRS	17.7% (44)
Forward-facing CRS	24.2% (60)
Booster seat	10.5% (26)
Seatbelt	43.1% (107)
No restraint	4.4% (11)
Frequency of being appropriately restrained while travelling in a motor vehicle	
Always	57.3% (142)
Almost always/Usually/Sometimes	37.1% (92)
Never	5.6% (14)
Seating position within the vehicle	
Front passenger seat	16.1% (40)
Rear seat (back seat of vehicle, 2nd or 3rd row of a minivan)	80.3% (199)
Someone’s lap	3.6% (9)

**Table 8:** Participants’ reasons for not ‘always’ using an appropriate restraint in a rideshare vehicle with their youngest child (*n* = 106).

Reasons	% (*n*)
Driver did not have a child restraint or booster seat available	39.6% (42)
Was only travelling a short distance	33.0% (35)
Was not required to use one in this situation	33.0% (35)
Did not have a child restraint or booster seat with me	26.4% (28)
Used a seatbelt instead of a child restraint or booster seat	23.6% (25)
Held child in my lap	9.4% (10)
Extra charge for a child restraint or booster seat	7.5% (8)
Did not want to carry around the child restraint or booster seat at the destination point	5.7% (6)
Driver had child restraint or booster seat available but preferred not to use it	4.7% (5)
Not sure	2.8% (3)
Other	0.0% (0)

**Table 9:** Participants’ experience with occupant restraint and regulations in rideshare vehicles (*n* = 130).

	% (*n*)
Who provided the child restraint or booster seat?	
Participant	74.6% (97)
Rideshare vehicle driver	25.4% (33)
Who installed the child restraint or booster seat in the rideshare vehicle?	
Participant	53.1% (69)
Rideshare vehicle driver	30.0% (39)
Participant and Rideshare vehicle driver	16.2% (21)
Rideshare company	0.8% (1)
Who adjusted the harness/seatbelt in the child restraint or booster seat in the rideshare vehicle?	
Participant	66.9% (87)
Rideshare vehicle driver	21.5% (28)
Participant and Rideshare vehicle driver	11.5% (15)
Confidence that the child restraint or booster seat was installed correctly?	
Very confident	40.8% (53)
Confident	45.4% (59)
Neither confident nor not confident	9.2% (12)
Not confident	4.6% (6)
Not at all confident	0.0% (0)
How confident are you that you followed your state or territory laws regarding the restraint of child occupants when using a rideshare service?	
Very confident	39.2% (51)
Confident	43.1% (56)
Neither confident nor not confident	13.8% (18)
Not confident	2.3% (3)
Not at all confident	1.5% (2)

**Table 10:** Characteristics of parents who have and have not travelled in a rideshare vehicle with their youngest child (*n* = 631).

	Have Travelled in a Rideshare Vehicle with Youngest Child *n* = 256 % (*n*)	Not Travelled in a Rideshare Vehicle with Youngest Child *n* = 375 % (*n*)	Significance
Participants’ socio-demographic characteristics			
Age (years)			
18–24	55.0% (22)	45.0% (18)	X^2^(4) = 12.30, *p* < 0.05, Cramer’s V = 0.14
25–34	42.4% (86)	57.6% (117)	
35–44	45.1% (78)	54.9% (95)	
45–54	34.8% (57)	65.2% (107)	
55+	25.5% (13)	74.5% (38)	
Sex			
Male	50.2% (116)	49.8% (115)	X^2^(1) = 14.06, *p* < 0.001, Phi =−0.15
Female	35.0% (140)	65.0% (260)	
Marital Status			
Single	45.3% (24)	54.7% (24)	X^2^(2) = 4.70, *p* = 0.10, Cramer’s V = 0.09
Married/Defacto	41.3% (224)	58.7% (318)	
Separated/Divorced/Widowed	23.5% (8)	76.5% (26)	
Highest level of completed education			
Primary/Intermediate/High	30.0% (30)	70.0% (70)	X^2^(2) = 19.74, *p* < 0.001, Cramer’s V = 0.18
Technical/Trade/Diploma	31.7% (60)	68.3% (129)	
Undergraduate/Postgraduate	48.5% (166)	51.5% (176)	
Yearly household income ($AUD) before taxes ^1^			
<$100,000	37.1% (143)	62.9% (242)	X^2^(1) = 7.04, *p* < 0.01, Phi = 0.11
>$100,000	48.2% (106)	51.8% (114)	
Residential state/territory ^2^			
Australian Capital Territory	70.6% (12)	29.4% (5)	X^2^(6) = 20.50, *p* < 0.01, Cramer’s V = 0.18
New South Wales	46.6% (90)	53.4% (103)	
Queensland	30.2% (35)	69.8% (81)	
South Australia	37.5% (15)	62.5% (25)	
Tasmania	36.4% (4)	63.6% (7)	
Victoria	44.1% (82)	55.9% (104)	
Western Australia	27.3% (18)	72.7% (48)	
Australian Capital Territory	70.6% (12)	29.4% (5)	
Participants’ driving characteristics			
Frequency of driving			
Daily	38.9% (138)	61.1% (217)	X^2^(2) = 2.23, *p* = 0.53, Cramer’s V = 0.06
4–6 times per week	41.7% (83)	58.3% (116)	
2–3 times per week	48.3% (29)	51.7% (31)	
<Once per week	35.3% (6)	64.7% (11)	
Estimated kms driven in the past year			
<5000 km	45.3% (58)	54.7% (70)	X^2^(2) = 4.71, *p* = 0.10, Cramer’s V = 0.09
5001–15,000 km	36.1% (106)	63.9% (188)	
>15,001 km	44.0% (92)	56.0% (117)	
Frequency of wearing a seatbelt			
Always	37.3% (218)	62.7% (366)	X^2^(1) = 34.17, *p* < 0.001, Phi = −0.23
Almost always/Usually Sometimes/Almost Never/Never	80.9% (38)	19.1% (9)	
Involved in a crash while driving?			
Yes	47.5% (28)	52.5% (31)	X^2^(1) = 1.28, *p* = 0.26, Phi = −0.05
No	39.7% (238)	60.3% (362)	
Involved in an at-fault crash while driving?			
Yes	58.1% (18)	41.9% (13)	X^2^(1) = 4.14, *p* < 0.05, Phi = −0.08
No	39.6% (218)	60.4% (333)	
Received a driving infringement?			
Yes	47.5% (38)	52.5% (42)	X^2^(1) = 1.83, *p* = 0.18, Phi = −0.05
Daily	38.9% (138)	61.1% (217)	
Participants’ youngest child’s characteristics			
Age (years)			
<1	12.1% (4)	87.9% (29)	X^2^(4) = 12.42, *p* < 0.05, Cramer’s V = 0.14
1–3	39.9% (73)	60.1% (110)	
4–7	42.1% (61)	57.9% (84)	
8–12	43.0% (61)	57.0% (81)	
13–17	44.5% (57)	55.5% (71)	
Sex ^3^			
Male	43.9% (150)	56.1% (192)	X^2^(1) = 3.27, *p* = 0.06, Phi = −0.07
Female	36.6% (105)	63.4% (182)	
Frequency of travelling in a vehicle			
Daily	37.3% (69)	62.7% (116)	X^2^(3) = 2.20, *p* = 0.53,Cramer’s V = 0.06
4–6 times per week	43.7% (107)	56.3% (138)	
2–3 times per week	41.1% (58)	58.9% (83)	
<Once per week	36.7% (22)	63.3% (38)	
Restraint type			
Rearward-facing CRS	28.2% (20)	71.8% (51)	X^2^(4) = 10.66, *p* < 0.05, Cramer’s V = 0.13
Forward-facing CRS	39.0% (55)	61.0% (86)	
Booster seat	44.5% (61)	55.5% (76)	
Seatbelt	40.9% (108)	59.1% (156)	
No restraint	66.7% (12)	33.3% (6)	
Frequency of restraint while travelling in a vehicle			
Always	35.2% (190)	64.8% (350)	X^2^(2) = 48.36, *p* < 0.001, Cramer’s V = 0.28
Almost always/Usually/Sometimes/Almost Never	78.5% (51)	21.5% (14)	
Never	57.7% (15)	42.3% (11)	

^1^ Participants who responded ‘Prefer not to say’ were excluded from this comparison. ^2^ Due to the small numbers of participants from the Northern Territory (*n* = 2), they were excluded from this comparison. ^3^ Due to the small numbers of children who identify as ‘Other’ (*n* = 2), they were excluded from the comparison.

## Data Availability

The dataset supporting the conclusions of this article is available from the corresponding author on reasonable request.

## Supplementary Materials

The following are available online at https://www.mdpi.com/article/10.3390/ijerph18178928/s1. Table S1: Child restraint requirements across Australian states and territories [20]. File S1: Parents’ attitudes towards using rideshare services to enhance children’s mobility.
