# Supplementary material for: What Are the Restraint Practices, Preferences, and Experiences When Australian Parents Travel with Their Children in a Rideshare Vehicle?
Source: Int J Environ Res Public Health. 2021 Aug 25;18(17):8928. doi: 10.3390/ijerph18178928 (PMC8431136; doi:10.3390/ijerph18178928)
Supplement: Supplementary file 1 [file ijerph-18-08928-s001.zip › ijerph-1336134-supplementary.pdf]

Supplementary Materials

What Are the Restraint Practices, Preferences, and Experiences When Australian Parents Travel with Their Children in a Rideshare Vehicle?

Table S1. Child restraint requirements across Australian states and territories [1].

State	National Transport Commission (National common law)	ACT	NSW	NT	QLD	SA	TAS	VIC	WA
Reference	Australian Road Rules	Road Transport (Road Rules) Regulation 2017	Road Rules 2014	Traffic Regulations 1999	Transport Operations (Road Use Management—Road Rules) Regulation 2009	Australian Road Rules (SA)	Road Rules 2019	Road Safety Road Rules 2017	Road Traffic Code 2000
Last Updated	22 November 2019	20 December 2019	29 November 2019	11 December 2019	28 June 2019	1 December 2019	1 December 2019	1 January 2020	1 January 2020
1. Requirements for children <6 months	Rearward-facing approved child restraint.	Equivalent to requirements stated in Australian Road Rules (NTC).	Equivalent to requirements stated in Australian Road Rules (NTC).	Refers to Australian Road Rules (NTC)	Equivalent to requirements stated in Australian Road Rules (NTC).	Equivalent to requirements stated in Australian Road Rules (NTC).	Equivalent to requirements stated in Australian Road Rules (NTC).	Equivalent to requirements stated in Australian Road Rules (NTC).	Equivalent to requirements stated in Australian Road Rules (NTC).

2. Requirements for children >6 months to <4 years	Either:<br><br>a) Rearward-facing approved child restraint, or<br><br>b) Forward-facing approved child restraint with inbuilt harness.	Equivalent to requirements stated in the Australian Road Rules (NTC).	Equivalent to requirements stated in the Australian Road Rules (NTC).		Equivalent to requirements stated in the Australian Road Rules (NTC).	Equivalent to requirements stated in the Australian Road Rules (NTC).	Equivalent to requirements stated in the Australian Road Rules (NTC).	Equivalent to requirements stated in the Australian Road Rules (NTC).	Equivalent to requirements stated in the Australian Road Rules (NTC).
3. Requirements for children >4 years to <7 years	Either:<br><br>a) Forward-facing approved child restraint with inbuilt harness, or<br><br>b) Approved booster seat & be restrained by either a suitable lap & sash type approved seatbelt or by a suitable approved child safety harness.<br>If seated in a seating position in a part of the vehicle that is designed primarily for the carriage of goods:	Equivalent to requirements stated in Australian Road Rules (NTC).	Equivalent to requirements stated in Australian Road Rules (NTC).		Equivalent to requirements stated in Australian Road Rules (NTC).	Equivalent to requirements stated in Australian Road Rules (NTC).	Equivalent to requirements stated in Australian Road Rules (NTC).	Passenger who is 4 years or older, but less than 7 years, must:<br><br>a) Be restrained in a suitable forward-facing approved child restraint with inbuilt harness; or<br><br>b) Occupy a seating position that is fitted with an approved seatbelt & be restrained in:	Either:<br><br>a) Forward-facing approved child restraint with an inbuilt harness, or<br><br>b) n approved booster seat & be restrained by either:<br><br>(i) a lap & sash type seatbelt, or<br><br>(ii) a lap only type seatbelt & (in relation to the upper body of the passenger) a child safety harness.

	i) be restrained by a suitable lap & sash type seatbelt, or<br><br>ii) have the mid-section of the child's body restrained by a suitable lap type seatbelt, & the upper body restrained by an approved child safety harness.							(i) a suitable lap & sash type approved seatbelt, or<br><br>(ii) a lap type seatbelt fitted with an approved child safety harness;<br><br>or (c) Be placed on a properly positioned approved booster seat & be restrained by either:<br><br>(i) a suitable lap & sash type approved seatbelt, or<br><br>(ii) a suitable approved child safety harness.	
4. Exemptions from requirements	Driver of a public minibus or taxi is exempt from requirements stated in (1), (2) & (3) if:<br><br>a) No suitable approved child	Driver of a public minibus, taxi or hire car is exempt from the requirements stated in (1), (2) & (3) if:	Provision of these requirements have effect in relation to passengers in or on taxis who are under 16 years,	Taxi drivers & drivers carrying passengers for hire or reward (commercial vehicle operators) do not have to provide	Driver of a booked hire vehicle or taxi is exempt from the requirements stated in (1), (2) & (3) if:	Driver of a public minibus or taxi is exempt from the requirements stated in (1), (2) & (3) if:	Driver of a taxi is exempt from the requirements stated in (1), (2) & (3) if:<br><br>a) No suitable approved child	If vehicle is a bus, public minibus, taxi or tow truck & there is no suitable approved child restraint or approved	Driver of a passenger transport vehicle is exempt from requirements stated in (1), (2), & (3) if:

	restraint available in the minibus or taxi for the passenger; &<br><br>b) Minibus or taxi has 2 or more rows of seats & the passenger is not in the front row of seats; &<br><br>c) No other law in the jurisdiction requiring all passengers in a minibus or taxi who are the same age as the passenger to be restrained in an approved child restraint.	a) No suitable approved child restraint available in the minibus, taxi or hire car for the passenger; &<br><br>b) If minibus, taxi or hire car has 2 or more rows of seats & the passenger is not in the front row of seats.	subject to the following modifications:<br><br>(a) Requirements stated in (2) applies only in relation to passengers who are 6 months or older, but less than 12 months.<br><br>b) Requirements stated in (3) does not apply to any passengers who are 4 years or older, but less than 7 years.	child restraints. However, they must ensure that:<br><br>a) An approved child restraint is used if available.<br><br>b) No passenger under four years sits in the front seat.<br><br>A child aged between 4 years & 7 years may only sit in the front row if all the other seats in the row or rows behind the front row are occupied by passengers who are also under 7 years.<br><br>All taxis should have an anchorage point that can be used to fit a child restraint;	a) No suitable approved child restraint available in the booked hire vehicle or taxi for the passenger; &<br><br>b) If booked hire vehicle or taxi has 2 or more rows of seats & the passenger is not in the front row of seats.	a) No suitable approved child restraint available in minibus or taxi for the passenger; &<br><br>b) If minibus or taxi has 2 or more rows of seats & the passenger is not in the front row of seats; &<br><br>c) No other law of this jurisdiction requiring all passengers in a minibus or taxi who are the same age as the passenger to be restrained in an approved child restraint.	restraint available in the taxi for the passenger; &<br><br>b) If taxi has 2 or more rows of seats & the passenger is not in the front row of seats; &<br><br>c) No other law of this jurisdiction requiring all passengers in a taxi who are the same age as the passenger to be restrained in an approved child restraint.	booster seat available in the motor vehicle for the passenger:<br><br>a) Passenger who is under 1 year is taken to be appropriately secured if the passenger is seated in the lap of another passenger who is 16 years or older; &<br><br>(b) Passenger who is at least 1 year but under 7 years is taken to be appropriately secured if the passenger is occupying a seating position fitted with a suitable approved seatbelt & wearing the seatbelt.	a) No suitable child restraint available in the vehicle for the passenger; &<br><br>b) Passenger is not in the front row of seats if the vehicle has 2 or more rows of seats.

				however, not all taxis will have a child restraint so you may need to provide your own.<br><br>Although an exemption will be provided for all commercial vehicle drivers, passengers must:<br><br>i) occupy individual seating positions & be appropriately restrained, &<br><br>ii) not occupy the same seating position as another passenger.					
5. Rideshare vehicles exempt?	No	No	No	Yes	Yes	No	No	No	Yes

File S1. Parents' attitudes towards using rideshare services to enhance children's mobility.

Please complete the following eligibility questions.

Are you aged 18 years or older?

○ Yes
○ No

Skip To: End of Survey If Are you aged 18 years or older? = No

Do you hold a valid driver's licence?

○ Yes
○ No

Skip To: End of Survey If Do you hold a valid driver's licence? = No

Are you an 'active' driver (i.e., at least once per week)? (Please think about the period pre-COVID19).

○ Yes
○ No

Skip To: End of Survey If Are you an 'active' driver (i.e., at least once per week)? (Please think about the period pre-COV... = No

Do you have at least one child (aged 17 years or younger) who lives with you and who regularly travels in a motor vehicle with you (i.e., at least once per week)? (Please think about the period pre-COVID19).

○ Yes
○ No

Skip To: End of Survey If Do you have at least one child (aged 17 years or younger) who lives with you and who regularly tr... = No

Do you currently live in Australia?

○ Yes
○ No

Skip To: End of Survey If Do you currently live in Australia? = No

End of Block: Eligibility questions

Start of Block: Demographics

Please complete the following demographic questions.

What is your age (years)?

____

What is your sex?

○ Male
○ Female
○ Other ____
○ Prefer not to say

What is your marital status?

○ Single
○ Married/Defacto
○ Separated/Divorced
○ Widowed
○ Other ____

What is your residential state or territory?

○ ACT
○ QLD
○ NSW
○ NT
○ SA
○ TAS
○ VIC
○ WA

What is your highest level of completed education?

○ Primary school
○ Intermediate (Year 10 equivalent)
○ VCE / HSC (Year 12 equivalent)
○ Technical / TAFE (including trade certificate / apprenticeship)
○ Diploma
○ Undergraduate degree
○ Postgraduate degree
○ Other ____

What is your current yearly household income (AUD), before taxes?

○ ≥$25,000
○ $25,001 - $50,000
○ $50,001 - $75,000
○ $75,001 - $100,000
○ $100,001 - $125,000
○ $125,001 - $150,000
○ $150,001 - $175,000
○ $175,001 - $200,000
○ $200,001 - $250,000
○ ≥$250,001
○ Prefer not to say

End of Block: Demographics

Start of Block: Child/Children

How many children do you have that currently live with you?

○ 1
○ 2
○ 3
○ 4
○ 5
○ 6
○ Other, please specify: ____

What is the age of your child(ren)? (years)

☐ Child 1 (oldest) ____
☐ Child 2 ____
☐ Child 3 ____
☐ Child 4 ____
☐ Child 5 ____
☐ Child 6 ____

End of Block: Child/Children

Start of Block: Youngest child

Please answer the remaining questions for your youngest child.

What is the sex of this child?

○ Male
○ Female
○ Other ____
○ Prefer not to say

How frequently does this child travel in a motor vehicle as a passenger with YOU as the driver? (Please think about the period pre-COVID19).

○ Daily
○ 4-6 days per week
○ 2-3 times a week
○ Once per week
○ Less than once per week
○ Less than once per month
○ Less than once per year
○ Never

What type of restraint does this child use most often when travelling in a motor vehicle as a passenger with YOU as the driver?

○ Rearward-facing child restraint
○ Forward-facing child restraint
○ Booster seat
○ Seatbelt
○ No restraint

How often does this child use a restraint while travelling in a motor vehicle as a passenger with YOU as the driver?

- ○ Always
- ○ Almost always
- ○ Usually
- ○ Sometimes
- ○ Almost never
- ○ Never

Where does this child sit most often when travelling in a motor vehicle as a passenger with YOU as the driver?

- ○ Front passenger seat
- ○ Rear seat (back seat of passenger vehicle, 2nd or 3rd row of minivan)
- ○ On someone's lap
- ○ Other - please specify: ____

End of Block: Youngest child

Start of Block: Rideshare service

How often does this child use other transportation modes with YOU?

	Daily	4-6 days per week	2-3 times per week	Once per week	Less than once per week	Less than once per month	Less than once per year	Never
Passenger in someone else's vehicle	○	○	○	○	○	○	○	○
Train	○	○	○	○	○	○	○	○
Bus	○	○	○	○	○	○	○	○
Tram	○	○	○	○	○	○	○	○
Rideshare service (e.g., Uber, Lyft, Shebah, etc.)	○	○	○	○	○	○	○	○
Taxi	○	○	○	○	○	○	○	○
Bicycle	○	○	○	○	○	○	○	○
Pedestrian	○	○	○	○	○	○	○	○
Other, please specify (If not applicable, please type NA and select 'Never'):	○	○	○	○	○	○	○	○

What are the reasons that you have not used a rideshare service to travel with this child? (Please select all responses that apply)

- ☐ Not available when I needed it
- ☐ Not convenient
- ☐ Not available in my area
- ☐ Too expensive
- ☐ Concerns over driver safety
- ☐ Concerns over vehicle safety
- ☐ No smartphone access
- ☐ Concerns over travelling with children
- ☐ Other (please specify): ____

What are the reasons for having concerns over travelling with children using a rideshare service? (Please select all responses that apply)

- ☐ Child required a child restraint or booster seat and I didn't have one with me
- ☐ Child required child restraint or booster seat and it wasn't provided by the driver
- ☐ Too many passengers for rideshare service vehicle
- ☐ Extra cost for child with child restraint or booster seat was too expensive
- ☐ Not a safe option when travelling with children
- ☐ Not a convenient option when travelling with children
- ☐ Not a practical option when travelling with children
- ☐ Other (please specify): ____

In what situations, and how frequently, have you used a rideshare service (e.g., Uber, Lyft, Shebah, etc.) to travel with this child over the past two years?

	Regularly used (more than 10 times)	Often used (6-10 times)	Occasionally used (2-5 times)	Rarely used (1 time)	Never used
For local travel during a holiday / out-of-town trip (e.g., airport to hotel, hotel to restaurant etc.)	○	○	○	○	○
For routine local travel where I live (e.g., daily activity, school drop off or pickup, shopping, social activities)	○	○	○	○	○
For non-routine local travel where I live (e.g., usual vehicle not available, emergencies, etc.)	○	○	○	○	○
To travel to a holiday / out-of-town trip destination	○	○	○	○	○

When you have used a rideshare service while travelling with this child, what position did this child sit in most frequently while travelling in the motor vehicle?

- ○ Front passenger seat
- ○ Rear side (left or right)
- ○ Rear centre
- ○ On someone's lap
- ○ Other - please specify: ____

When you have used a rideshare service while travelling with this child, how often was an appropriate restraint used (e.g., child restraint, booster seat or seatbelt based on child's age/size)?

○ Always
○ Almost always
○ Usually
○ Sometimes
○ Almost never
○ Never

What was the reason that an appropriate restraint was not used? (Please select all responses that apply)

☐ Was only travelling a short distance
☐ Did not have my child restraint or booster seat with me
☐ Driver did not have a child restraint or booster seat available
☐ Was not required to use one in this situation
☐ Used a seatbelt instead of a child restraint or booster seat
☐ Held child in my lap
☐ Driver had child restraint or booster seat available but preferred not to use it
☐ Extra charge for a child restraint or booster seat
☐ Did not want to carry around the child restraint or booster seat at the destination point
☐ Not sure
☐ Other (please specify): ____

When you have used a rideshare service while travelling with this child, what type of restraint was used by this child?

○ Rearward-facing child restraint
○ Forward-facing child restraint
○ Booster seat
○ Seatbelt
○ No restraint

When you have used a rideshare service while travelling with this child, who provided the child restraint or booster seat?

○ You provided the child restraint or booster seat
○ Driver provided the child restraint or booster seat

When you have used a rideshare service while travelling with this child, who installed the child restraint or booster seat?

○ You
○ Driver
○ You and driver
○ Other (please specify): ____

When you have used a rideshare service while travelling with this child, who adjusted the harness in the child restraint or booster seat?

○ You
○ Driver
○ You and driver
○ Other (please specify): ____

When you have used a rideshare service while travelling with this child, how confident were you that the child restraint or booster seat was correctly installed?

○ Very confident
○ Confident
○ Neither confident or not confident
○ Not confident
○ Not at all confident

How confident are you that you followed your state or territory laws regarding the restraint of child occupants when using a rideshare service?

○ Very confident
○ Confident
○ Somewhat confident
○ A little confident
○ Not at all confident
○ Not applicable because have not used a rideshare service with my child

End of Block: Rideshare service

Start of Block: Driving and licensing

Please complete the following driving and licensing questions.

Are there any conditions or restrictions on your driver's licence? (e.g., wearing corrective lenses)

○ Yes (If yes, please specify conditions or restrictions.) ____
○ No

At what age did you obtain your licence to drive unsupervised? (years)

____

What is the make of the vehicle that you drive most often? (e.g., Toyota, Mazda, Holden)

____

What is the model of vehicle you drive most often? (e.g., Golf, i30, Camry)

____

What is the year of manufacture of the vehicle that you drive most often (if known)? (e.g., 2000, 2006)

____

How frequently do you drive your vehicle? (Please think about the period pre-COVID19).

○ Daily
○ 4-6 times per week
○ 2-3 times a week
○ Once per week
○ Less than once per week

Please estimate the number of kilometres you have driven in your motor vehicle over the past year? (If you are not sure, please estimate. For example, if your vehicle does approximately 500km/tank and you fill your tank every second week, you have driven approximately 13,000 km/year). (Please think about the period pre-COVID19).

○ ≤ 1,000km
○ 1,001-3,000km
○ 3,001-5,000km
○ 5,001-10,000km
○ 10,001-15,000km
○ 15,001-20,000km
○ 20,001-25,000km
○ ≥25,001km

Over the past two years, have you been involved in a crash while YOU were driving (including minor crashes)? (If 'Yes', please indicate how many crashes?)

○ Yes ____
○ No

Over the past two years, have you been involved in an at-fault crash while YOU were driving (including minor crashes)? (If 'Yes', please indicate how many crashes?)

○ Yes ____
○ No

Over the past two years, have you been involved in a crash (including minor crashes) that resulted in injuries to you or someone else while YOU were driving? (Tick all that apply, and indicate how many crash-related injuries?)

☐ No injuries to anyone ____
☐ Minor injuries (i.e., not hospitalised) to you or someone else ____
☐ Serious injuries (i.e., hospitalised) to you or someone else ____
☐ Fatal injuries to someone else ____

What type of crash(es) were you involved in while YOU were driving? (Tick all that apply, and indicate how many?)

☐ Crash with a pedestrian/cyclist ____
☐ Frontal impact: The front of your vehicle impacted against / was impacted by another vehicle or obstacle (not including a pedestrian/cyclist) ____
☐ Rear impact: The rear of your vehicle impacted against / was impacted by another vehicle or obstacle (not including a pedestrian/cyclist) ____
☐ Side impact: The side of your vehicle impacted against / was impacted by another vehicle or obstacle (not including a pedestrian/cyclist) ____
☐ Other: ____

Over the past two years, have YOU been cited for speeding? (If 'Yes', please indicate how many citations?)

○ Yes: ____
○ No

Over the past two years, have YOU been cited for failing to stop at a stop sign or traffic signal (including red light cameras)? (If 'Yes', please indicate how many citations?)

○ Yes: ____
○ No

Over the past two years, have YOU been cited for any other driving offences (other than a parking ticket)? (If 'Yes', please specify the driving offence(s) and how many?)

○ Yes: ____
○ No

How often do YOU wear your seatbelt while travelling in a motor vehicle?

○ Always
○ Almost always
○ Usually
○ Sometimes
○ Almost never
○ Never

Start of Block: Survey link

If you would like to enter the draw to win one of five $100 gift vouchers, please click on the link below which will guide you to a separate form where you can provide your contact details, as well indicate your willingness to be contacted about participating in follow-up research, please click here.

End of Block: Survey link

References

1. National Transport Commission. Road Transport Legislation—Australian Road Rules. 2019. Available online: http://https://www.legislation.gov.au/Details/F2016C00534 (accessed on 10 June 2021).
